# Dietary Tomato Consumption and the Risk of Prostate Cancer: A Meta-Analysis

**DOI:** 10.3389/fnut.2021.625185

**Published:** 2021-05-04

**Authors:** Jie Luo, Dandan Ke, Qingwei He

**Affiliations:** Department of Urology, The First Affiliated Hospital, Zhejiang University School of Medicine, Zhejiang, China

**Keywords:** prostate cancer, tomato, cohort, meta-analysis, risk

## Abstract

**Objective:** Several epidemiological studies have linked tomato products consumption with prostate cancer risk; however, the findings yielded inconsistent results. The aim of the present meta-analysis is to summary the evidence on this association based on eligible cohort studies.

**Materials and Methods:** A comprehensive literature search of articles was performed in March 2021 using PubMed, ISI Web of Science, and Scopus databases. A random-effects model was used to calculate the combined relative risks (RRs) and their corresponding 95% confidence intervals (CIs). Heterogeneity across studies was assessed using Cochran's Q statistic and the *I*^2^ score.

**Results:** A total of 10 prospective studies were finally included in our meta-analysis. There was no evidence of a significant association between tomato products consumption and prostate cancer risk (RR 0.91, 95% CI 0.79–1.03, *P* = 0.138). Subgroup meta-analyses were performed by tomato types, geographical region, publication year, study quality and number of cases. No significant associations were observed in any subgroups (all *P* > 0.05). No significant publication bias was observed using Begg's test (*P* = 0.602) or Egger's test (*P* = 0.957).

**Conclusion:** The results of this meta-analysis indicated that tomato consumption was not related with the risk of prostate cancer. Further prospective large-scale cohort studies are still warranted to verify our findings.

## Introduction

Prostate cancer is the second most common cancer diagnosis made in men and the fifth leading cause of death worldwide. According to GLOBOCAN 2018 estimates, about 1.27 million new cases of prostate cancer occurred worldwide in 2018, with higher prevalence in the developed world compared with that in the developing countries (1). Prostate cancer risk has been reported to be positively associated with the following: black ethnicity, having a family history of prostate cancer, and advanced age (2). Dietary habits, including nutrients and dietary patterns, potentially affect prostate cancer pathogenesis and progression through various mechanisms mediated inflammation, antioxidant effects, and the action of sex hormones (3). Several dietary factors (4, 5) have been identified to be associated with the risk of prostate cancer, although controversial results have been reported for almost all nutrients.

Dietary tomato or lycopene intake has been shown beneficial for multiple health outcomes in humans (6). The association between tomato consumption and prostate cancer risk also has been assessed by several observational studies and meta-analyses. An early meta-analysis by Xu et al. (7) published in 2016 included a total of 24 case-control and cohort studies with 15,099 cases and reported that tomato intake may have a weak protective effect against prostate cancer. A dose-response meta-analysis published in 2018 also reported that increased tomato consumption was inversely associated with prostate cancer risk (8). However, a recent large pooled analysis failed to find a protective role of tomato consumption on prostate cancer (9). Considering these inconsistencies, we performed the present meta-analysis based on all eligible cohort studies to re-evaluate the relationship between tomato intake and the risk of prostate cancer.

## Materials and Methods

### Literature Search

Literature search was performed in March 2021 using PubMed, ISI Web of Science, and Scopus databases by two independent reviewers (JL and DK) with the following search algorithm: (tomato or tomatoes or lycopene or vegetable or vegetables or diet or nutrition) and (prostate cancer or prostatic cancer or prostate neoplasm or prostatic neoplasm) and (cohort or case-cohort or nested case-control or prospective or trial). The reference lists of retrieved articles and reviews were also examined to identify any additional relevant studies. This systematic review and meta-analysis was designed, performed, and reported according to the standards of quality for reporting meta-analyses (10).

### Inclusion Criteria

An included study met all the following criteria: (i) the risk factor was consumption of tomato or tomato products; (ii) the outcome was the incidence of prostate cancer; (iii) study design was cohort, case-cohort, nested case-control or clinical trial; and (*iv*) the risk estimates with their corresponding 95% confidence intervals (CIs) were provided. If multiple studies used data from the same population, the study with the largest sample size was included in this meta-analysis. There were no restrictions on publication language, publication date or publication status.

### Methodological Quality Assessment

Methodological quality of each included study was assessed using Newcastle-Ottawa Scale (NOS, http://www.ohri.ca/programs/clinical_epidemiology/oxford.asp) by two independent reviewers (JL and DK). NOS is a tool used for assessing the quality of non-randomized studies included in a systematic review and/or meta-analyses. NOS contains eight items within three domain and the highest score is 9. A study with score from 7–9 was considered as high quality.

### Data Collection

Data was extracted and recorded using a pre-defined form by two independent reviewers (JL and DK). The following data were collected from each study: first author's surname, publication year, country where the study was performed, study design, age, number of cases, method of exposure measurement, method of outcome assessment, fully-adjusted risk estimates with their corresponding 95% CIs, and adjusted variables in the statistical analysis. If data were not reported in the primary study, the items were designated “not applicable.” Any discrepancies were resolved by consensus.

### Statistical Methods

Two studies (11, 12) provided the relative risks (RRs) for raw tomato and cooked tomato separately. In this situation, the overall RR was calculated by combined these risk estimates using a fixed effect model with inverse-variance method (13). Finally, the summary RR of all included studies was calculated using a DerSimonian and Laird random-effects model (14), which incorporates both within- and between-study variability. Stratified analyses were performed based on tomato types, study region, publication year, study quality, and sample size. One duplicate publication (15) was excluded from the main analysis but was included in subgroup analysis. Significant heterogeneity across studies was detected based on *Q* statistic (significant level set at 0.1) (16). The *I*^2^ score was used to assess the degree of heterogeneity (*I*^2^ <25%: small heterogeneity; *I*^2^ = 25–50%: moderate heterogeneity; *I*^2^ > 50%: large heterogeneity). A sensitivity analysis was conducted by excluding each study in turn and repeated the meta-analysis to assess the impact of each included study on the summary risk estimate. Publication bias was evaluated using Begg's test (17) and Egger's test (18).

Dose–response meta-analysis was performed with the method described by Greenland et al. (19) and Orsini et al. (20). Only studies that provided at least 3 quantitative categories were included. When a range of tomato consumption was provided, the median or mean value was regarded as the corresponding exposure dose. If the median or mean value was not reported, we used the midpoint of each category. If the lower or upper boundary was not provided, the boundary was assumed to have the same amplitude as the adjacent category. We converted the amount of tomato intake into a uniform measurement of grams (g) per day with the following equivalencies: 148 g per serving for raw tomatoes; 60 g per serving for cooked tomatoes; 104 g per serving (1:1 ratio of raw and cooked tomato products) for tomato products (8). A potential non-linear dose–response relationship between tomato intake and prostate cancer risk was examined using restricted cubic splines with three knots at the 25th, 50th, and 75th percentiles of the distribution. A *P*-value for non-linearity was calculated by testing the null hypothesis that the coefficient of the second spline was equal to zero (21). All statistical analyses were performed using STATA 11.0 (StataCorp, College Station, TX) with two-sided *P*-values (set at 0.05).

## Results

### Study Search and Main Characteristics of Included Studies

**Figure 1:**
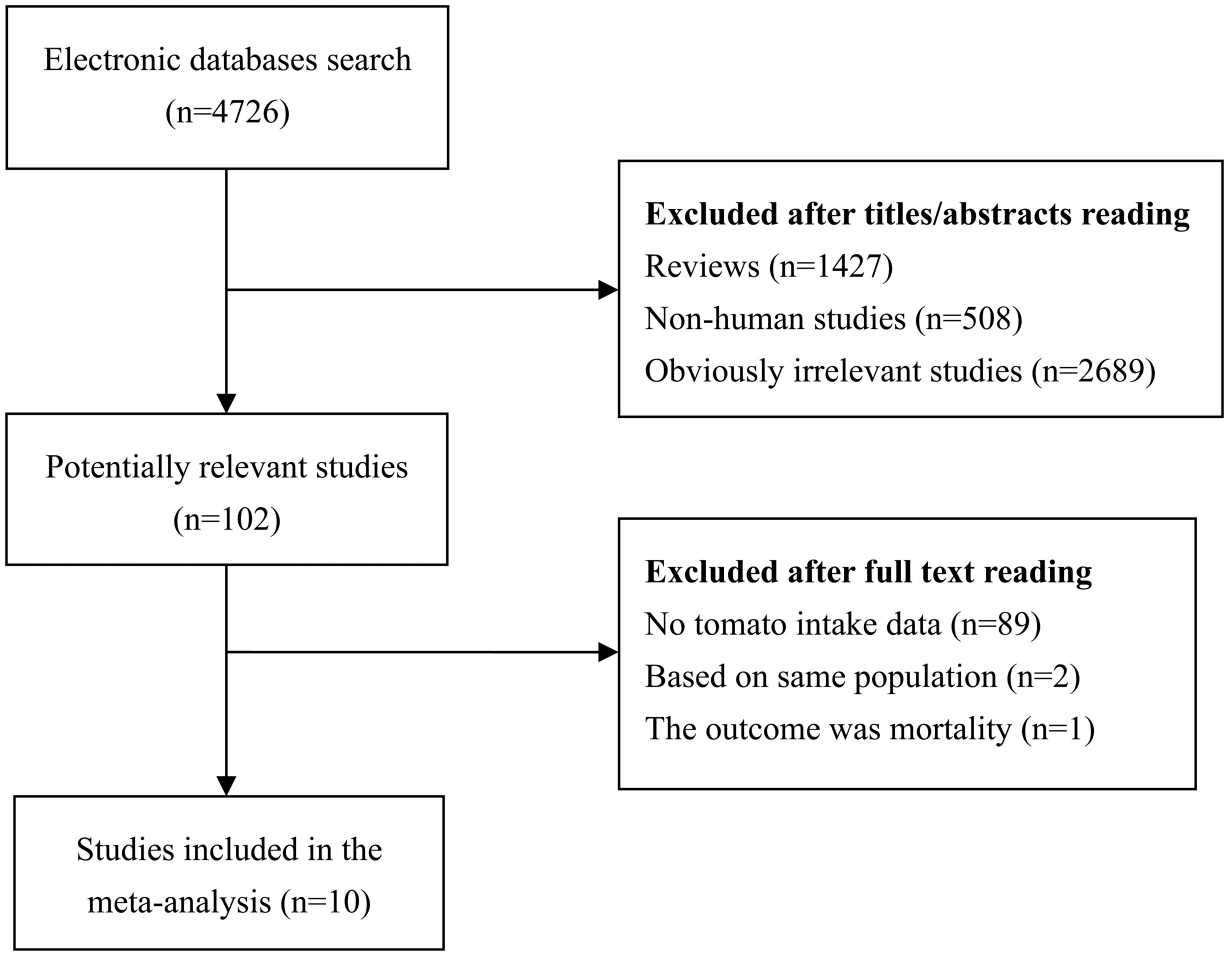
A systematic literature search and review.

The detailed process of literature search and review has been shown in Figure 1. Initial search identified 4,726 potentially relevant studies. After reading titles or abstracts, 1,427 reviews, 508 non-human studies and 2,689 obviously irrelevant studies were removed. After further full text reading, 92 studies were removed with the following reasons: no tomato intake data (*n* = 89), based on same population (*n* = 2), and the outcome was mortality (*n* = 1). A total of 10 prospective studies (11, 12, 15, 22–28) were finally included in our meta-analysis. These studies were published between 1989 and 2020 and from the following geographical region: Europe (*n* = 1), North America (*n* = 7), Asia (*n* = 1), and Oceania (*n* = 1). Exposure data was collected by self-administrated questionnaire, except for the Diallo et al.'s study (22), which used interview. Outcome data was confirmed histologically or collected from cancer registry/medical records. Study quality scores assessed by NOS ranged from 5 to 8, with a mean value of 6.7. The main study characteristics have been summarized in Table 1.

**Table 1:** Main characteristics of the included studies.

**References**	**Country**	**Study name**	**Age (y)**	**No. of cases**	**Follow-up (y)**	**Exposure assessment**	**Tomato types**	**Outcome assessment**	**Adjusted variables**	**NOS** **score**
Fraser et al. (12)	USA and Canada	AHS-2	30–104	1,226	7.9	Questionnaire	Raw; processed	Cancer registry	Age, family history, race, education, smoking, BPH history, PSA screening history, dairy consumption, energy intake, and being vegan	7
Graff et al. (15)	USA	HPFS	40–75	5,543	23	Questionnaire	Tomato sauce	Medical records	Age, family history, calendar time, race, height, BMI at age 21 y, current BMI, physical activity, smoking, diabetes, PSA testing, use of multivitamins, total calories, and intakes of calcium, a-linolenic acid, supplemental vitamin E, alcohol, and coffee	8
Diallo et al. (22)	France	SU.VI.MAX cohort	NA	139	12.6	Interview	Tomato products	Biopsy	Age, family history, smoking, education, physical activity, height, BMI, alcohol, energy intake, intervention group of the initial SU.VI.MAX trial, number of 24-h dietary records, plasma PSA, Ca intake, dairy product intake and plasma α-tocopherol and Se concentrations	8
Er et al. (23)	United Kingdom	ProtecT trial	50–69	1,806	2001–2009	Questionnaire	Tomato products	Histologically confirmed	Age, family history, smoking, recruitment center, and energy intake	6
Takachi et al. (28)	Japan	JPHC Study	45–74	339	1995–2004	Questionnaire	Tomatoes and tomato products	Cancer registry	Age, BMI, smoking, public health center area, alcohol, dairy food, soy products, green tea, vitamin supplement use, marital status, screening examination	6
Ambrosini et al. (11)	Australia	CARET	NA	97	1990–2004	Questionnaire	Raw; cooked	Cancer registry	Age, fruit and vegetable intake, randomly assigned retinol or β-carotene supplement, and source of crocidolite exposure	6
Kirsh et al. (25)	USA	PLCO	55–74	1,338	4.2	Questionnaire	Raw; processed	Medical/pathologic records	Age, family history, race, study center, BMI, smoking, physical activity, energy intake, supplemental vitamin E, total fat, red meat, diabetes, aspirin use, and previous number of screening exams	7
Stram et al. (27)	USA	MEC	45–75	3,922	7	Questionnaire	Raw; processed	SEER registry	Age, family history, BMI, and education	7
Giovannucci et al. (24)	USA	HPFS	40–75	812	1986–1992	Questionnaire	Tomato products	Medical records	Age and energy intake	7
Mills et al. (26)	USA	AHS-1	NA	180	6	Questionnaire	Tomatoes	Histologically confirmed	Age, education, current use of meat, poultry, or fish, current fish only, beans, legumes or peas, citrus fruit, dry fruit, and index of fruit, nuts	5

### Summary Analysis and Study Heterogeneity

**Figure 2:**
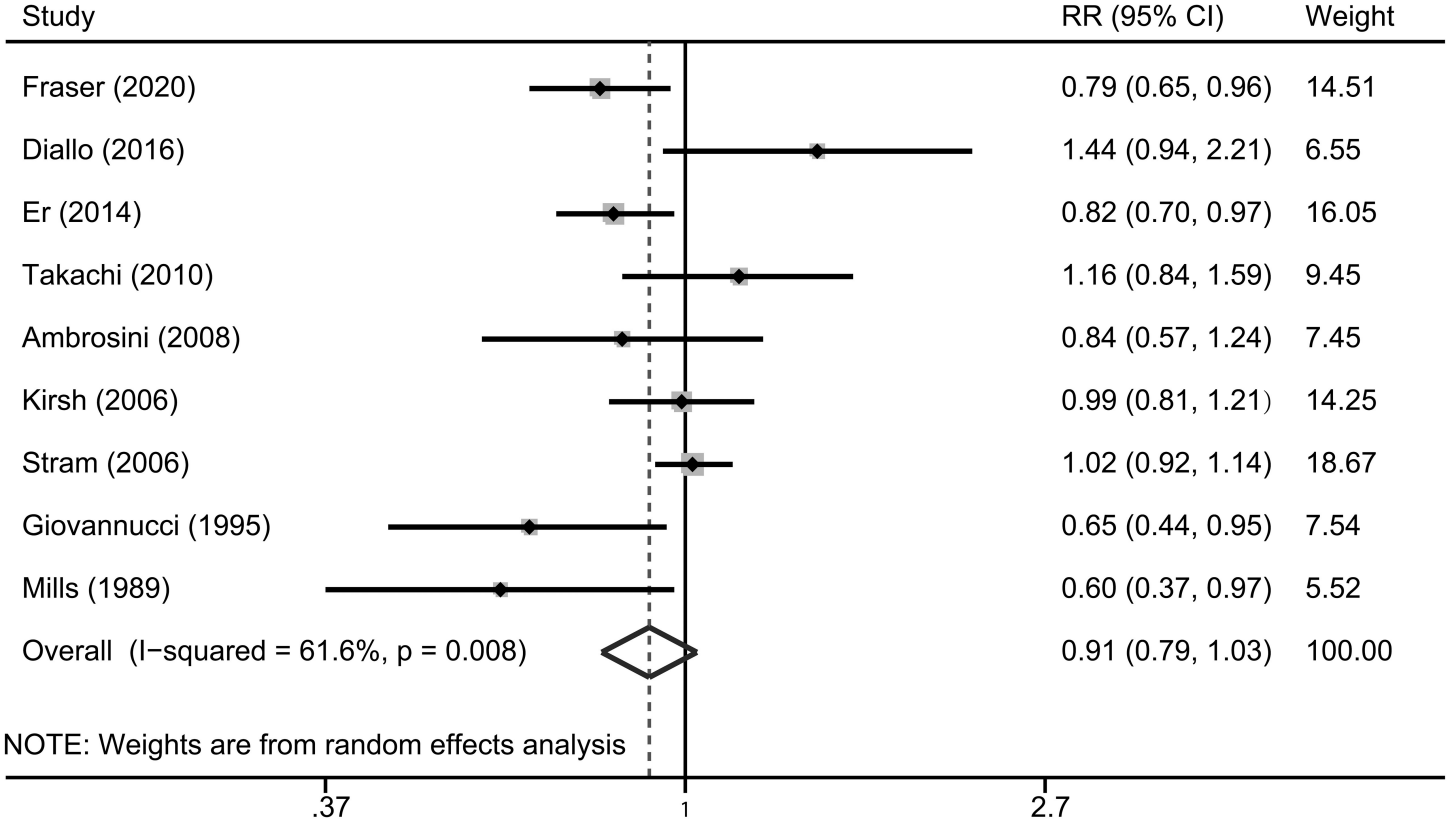
A forest plot showing risk estimates of the association between tomato consumption and prostate cancer risk.

Multivariable adjusted RRs with their CIs for each included study and for the combination of all studies are presented in Figure 2. The highest consumption of tomato products was not significantly associated with the risk of prostate cancer, compared with the lowest consumption group (RR 0.91, 95% CI 0.79–1.03, *P* = 0.138). There was significant heterogeneity across included studies (*P* = 0.008 for heterogeneity, *I*^2^ = 61.6%).

### Subgroup Analysis

Subgroup meta-analyses were performed by tomato types, geographical region, publication year, study quality and number of cases. No significant associations were observed in any subgroups (Table 2, all *P* > 0.05).

**Table 2:** Subgroup analysis of tomato intake with prostate cancer risk.

**Subgroup**	**Included studies**	**Pooled RR (95% CI)**	***P***	**Heterogeneity**		
				**Q**	***I*^**2**^ (%)**	***P***
Total	9	0.91 (0.79–1.03)	0.138	20.83	61.6	0.008
**Tomato types**						
Raw	4	0.96 (0.88–1.05)	0.378	1.37	0.0	0.712
Cooked	4	0.91 (0.75–1.10)	0.305	8.54	64.9	0.036
Sauce	3	0.96 (0.82–1.14)	0.666	8.96	77.7	0.011
Juice	4	0.98 (0.91–1.05)	0.560	2.48	0.0	0.479
**Geographical region**						
North America	5	0.85 (0.71–1.02)	0.081	12.39	67.7	0.015
Europe	2	1.05 (0.61–1.81)	0.866	5.82	82.8	0.016
Asia	1	1.16 (0.84–1.59)	0.362	–	–	–
Oceania	1	0.84 (0.57–1.24)	0.379	–	–	–
**Publication year**						
≥2010	4	0.96 (0.76–1.20)	0.716	9.88	69.6	0.020
<2010	5	0.87 (0.73–1.05)	0.145	9.36	57.3	0.053
**Study quality**						
High (NOS ≥ 7)	5	0.94 (0.78–1.12)	0.462	12.55	68.1	0.014
Low (NOS <7)	4	0.86 (0.69–1.07)	0.167	5.84	48.7	0.119
**No. of cases**						
≥1,000	4	0.91 (0.79–1.04)	0.155	8.20	63.4	0.042
<1,000	5	0.90 (0.65–1.23)	0.494	12.61	68.3	0.013

*NOS, Newcastle-Ottawa scale; No, number; RR, relative risk; CI, confidence interval*.

### Sensitivity Analysis and Publication Bias Analysis

**Figure 3:**
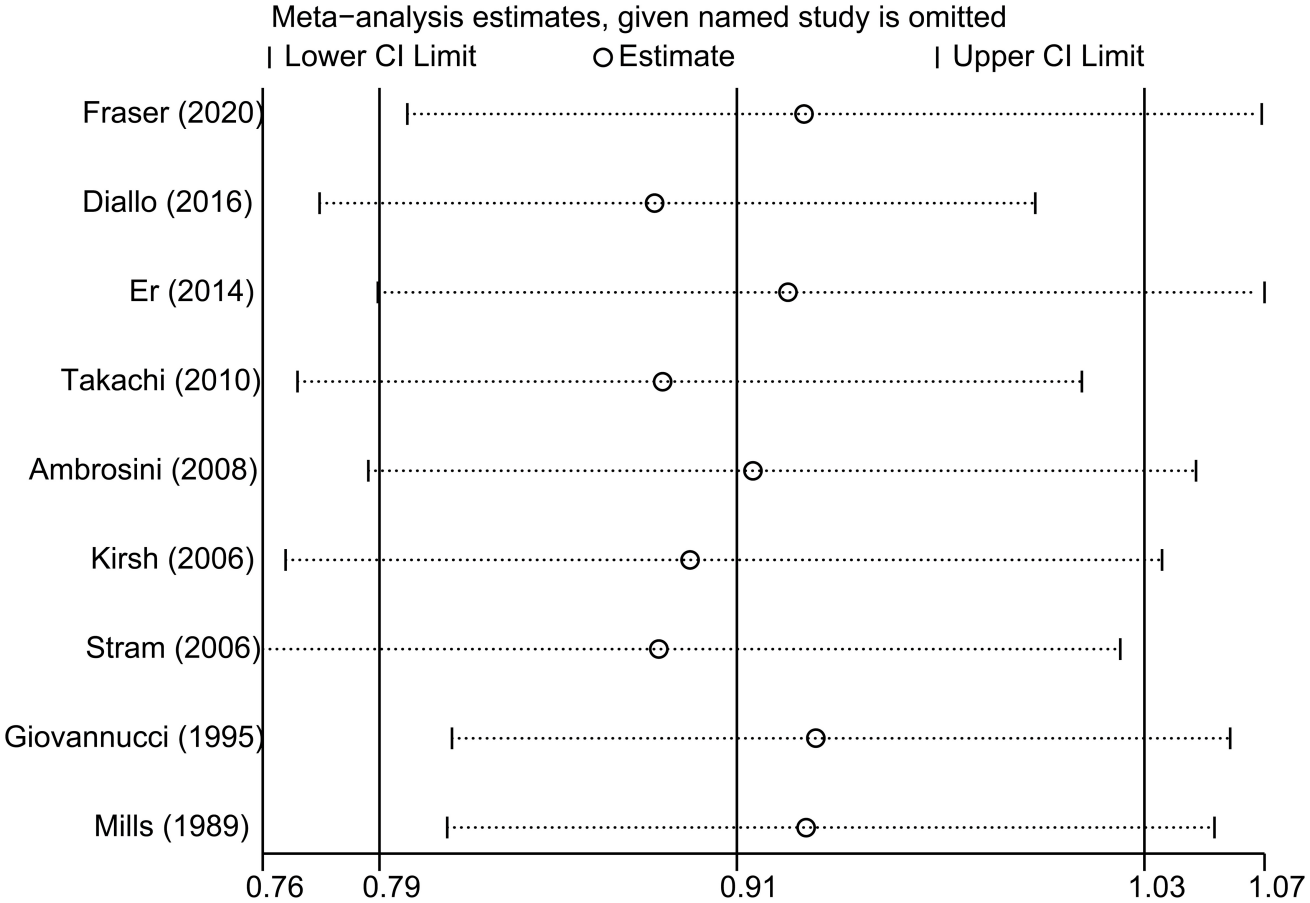
Sensitivity analysis was conducted by omitting each study in turn and repeated the meta-analysis.

**Figure 4:**
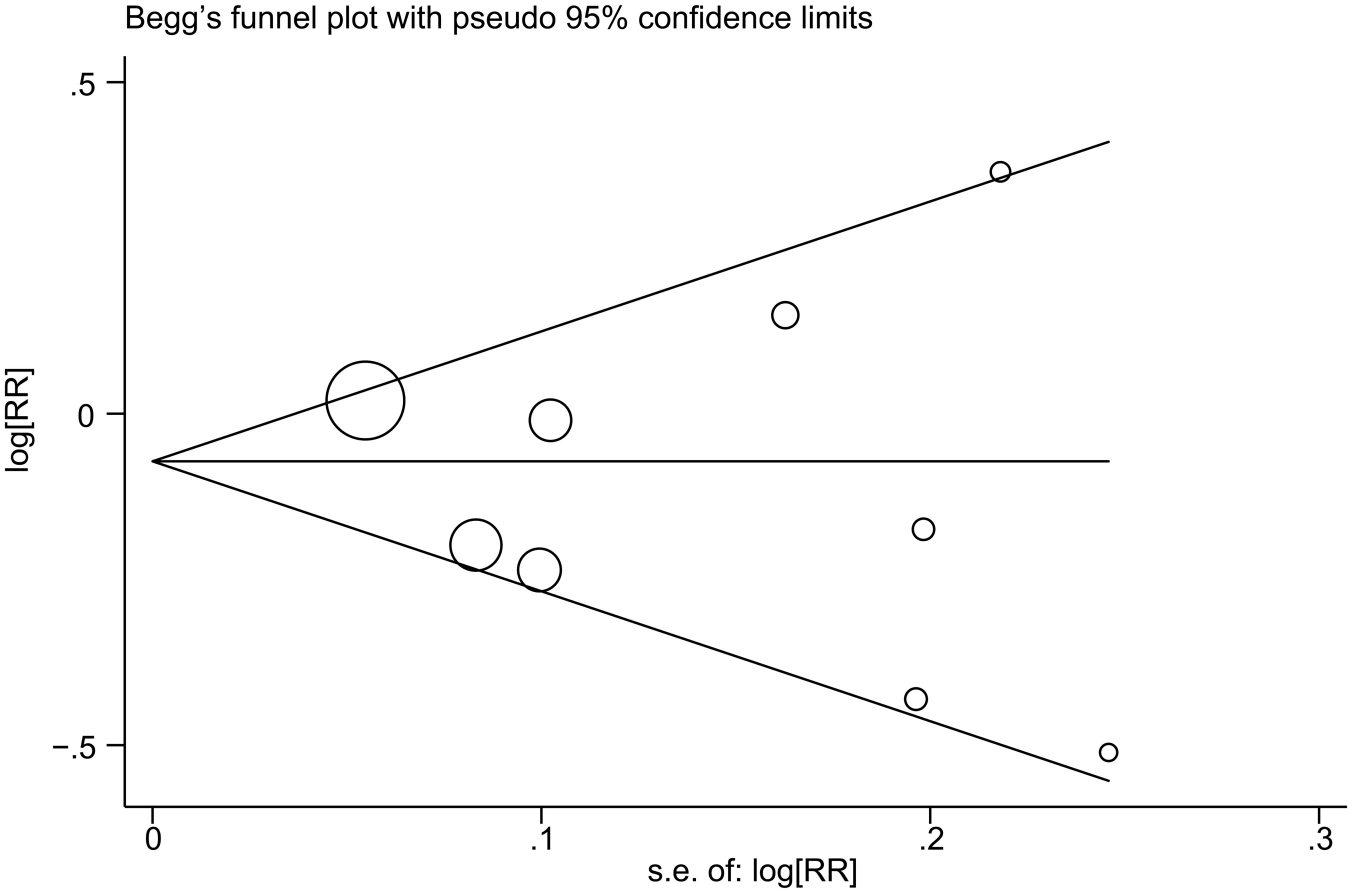
Publication bias was assessed with a Begg's funnel plot.

The impact of individual study on the summary RR was assessed by repeating the meta-analysis after removing each study in turn. The study-specific RRs ranged from a low of 0.88 (95% CI 0.78–1.00) to a high of 0.93 (95% CI 0.80–1.07) by removing the study by Diallo et al. (22) and the study by Fraser et al. (12), respectively (Figure 3). Similar results were obtained when excluding two studies (11, 12) that reported the RRs for raw tomato and cooked tomato separately (pooled RR = 0.93, 95% CI 0.80–1.09). No significant publication bias was observed using Begg's test (*P* = 0.602, Figure 4) or Egger's test (*P* = 0.957).

### Dose-Response Meta-Analysis for the Association Between Tomato Intake and Prostate Cancer Risk

**Figure 5:**
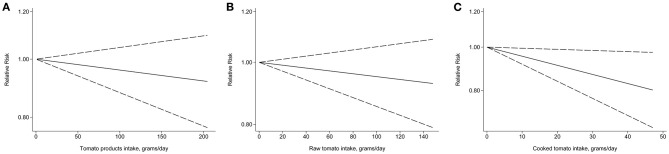
Relative risk for prostate cancer by doses of tomato intake based on the results of the dose–response meta-analyses. Solid line represents the estimated relative risks and the dotted lines represent the 95% confidence intervals. **(A)** Tomato products; **(B)** Raw tomato; **(C)** Cooked tomato.

The number of studies eligible for the dose–response analysis was five (22, 24–26, 28), four (11, 12, 24, 25), and thee (11, 12, 25) for tomato products, raw tomatoes, and cooked tomatoes, respectively. There was no evidence of non-linearity. All *P*-values for non-linear assessment were >0.05. The pooled RRs for a 20 g/day increase in tomato intake were 0.99 (95% CI 0.97–1.01), 0.99 (95% CI 0.97–1.01), and 0.98 (95% CI 0.96–1.00), respectively (Figure 5).

## Discussion

The current meta-analysis systematically summarized the results of 10 cohort studies including a total of 15,402 cases. The pooled results of this study indicated that the consumption of tomato products was not associated with the risk of prostate cancer. In addition, no significant associations were observed in any individual tomato products, including raw tomatoes, cooked tomatoes, tomato sauces, and tomato juice, in subgroup analyses. Although a borderline significant association between cooked tomato intake and prostate cancer risk was observed in dose-response analysis, this result should be interpreted with caution as only three studies were eligible and the findings may have been due to chance.

The findings of our study were not completely consistent with the previous meta-analyses (7, 8, 29, 30). The earliest meta-analysis by Etminan et al. (29) published in 2004 found that higher consumption of raw tomato or cooked tomato products was significantly related with a lower risk of prostate cancer. The study by Xu et al. (7) analyzed a total of 24 observational studies and reported that tomato intake was associated with a reduced risk of prostate cancer (RR 0.86, 95% CI 0.75–0.98, *P* = 0.019). The mostly recent meta-analysis published in 2018 (8) suggested a dose-response relationship with prostate cancer risk for total tomato consumption and for cooked tomatoes and sauces based on thirty observational studies. It worth to mention that the majority of studies included in these meta-analyses were case-control studies, which were prone to selection and recall bias. The evidence from cohort studies on the association of tomato intake with prostate cancer risk was relatively weak. For example, in the subgroup analysis of Xu et al.'s study (7), the RR for cohort studies was 0.96 (95% CI 0.84–1.10, *P* = 0.579). An early meta-analysis based on three prospective studies also found no significant association between raw or cooked tomato intake and prostate cancer risk (30).

One of the main mechanisms that has been proposed to explain the favorable effect of tomato intake on cancer prevention involves lycopene, which are found in high amounts in tomato products (31). A recent study indicated that lycopene is a promising chemotherapy drug by inhibiting prostate cancer progression via the inflammatory response signaling (32). Wang et al. (33) found that dietary lycopene consumption and its blood level were all associated with the risk of prostate cancer based on a systematic review and dose-response meta-analysis. Grainger et al. (34) performed a phase 2 dose-escalating study and firstly confirmed the phytoene and phytofluene in prostate tissue after a dietary intervention. However, Rowles et al. (35) found that tomato or lycopene had no significant impact on the emergence of castration-resistant prostate cancer in a murine model. Therefore, the role of tomato and lycopene intake on the incidence of prostate cancer is still inconsistent. Our meta-analysis based on published cohort studies supported that there was no clear association between tomato consumption and prostate cancer risk.

Our meta-analysis has several strengths. First, the present study had large sample size and thus enhanced the statistical power. Second, this meta-analysis only included cohort studies, which avoided the recall and selection biases. Third, based on the NOS scores, the methodological quality of the included studies was generally high. Finally, no obvious publication bias was observed across studies, indicating that the results were less likely to prone to biases.

Several limitations should also be acknowledged. First, the number of eligible studies was relatively limited and most of the included studies were performed in USA, which limited the generality of the findings of our study. Second, a certain degree of heterogeneity existed, which may distort the summary risk estimate. Finally, the types of tomato product and the cut-off points were various among the included studies, which might also impact the current analysis.

In conclusion, the results of this study indicated that tomato consumption was not related with the risk of prostate cancer. Further prospective large-scale cohort studies are still warranted to verify our findings.

## Data Availability Statement

The original contributions presented in the study are included in the article/supplementary material, further inquiries can be directed to the corresponding author/s.

## Author Contributions

JL and DK contributed to the conception or design of the work. JL, DK, and QH contributed to the acquisition, analysis, or interpretation of data for the work. JL and DK drafted the manuscript. QH critically revised the manuscript. All authors gave final approval and agree to be accountable for all aspects of work ensuring integrity and accuracy.

## Conflict of Interest

The authors declare that the research was conducted in the absence of any commercial or financial relationships that could be construed as a potential conflict of interest.
